# Schizophrenia proteomics: biomarkers on the path to laboratory medicine?

**DOI:** 10.1186/1746-1596-1-11

**Published:** 2006-07-17

**Authors:** Shaheen Emmanuel Lakhan

**Affiliations:** 1 Global Neuroscience Initiative Foundation, Los Angeles, CA, USA

## Abstract

Over two million Americans are afflicted with schizophrenia, a debilitating mental health disorder with a unique symptomatic and epidemiological profile. Genomics studies have hinted towards candidate schizophrenia susceptibility chromosomal loci and genes. Modern proteomic tools, particularly mass spectrometry and expression scanning, aim to identify both pathogenic-revealing and diagnostically significant biomarkers. Only a few studies on basic proteomics have been conducted for psychiatric disorders relative to the plethora of cancer specific experiments. One such proteomic utility enables the discovery of proteins and biological marker fingerprinting profiling techniques (SELDI-TOF-MS), and then subjects them to tandem mass spectrometric fragmentation and *de novo *protein sequencing (MALDI-TOF/TOF-MS) for the accurate identification and characterization of the proteins. Such utilities can explain the pathogenesis of neuro-psychiatric disease, provide more objective testing methods, and further demonstrate a biological basis to mental illness. Although clinical proteomics in schizophrenia have yet to reveal a biomarker with diagnostic specificity, methods that better characterize the disorder using endophenotypes can advance findings. Schizophrenia biomarkers could potentially revolutionize its psychopharmacology, changing it into a more hypothesis and genomic/proteomic-driven science.

## Schizophrenia proteomics: biomarkers on the path to laboratory medicine?

Affecting nearly 1% of the world's population, schizophrenia is marked by chronic psychosis and social, occupational, behavioral, and cognitive impairment. This debilitating psychiatric disorder requires a disproportionate share of medical resources due to its early onset and chronic and severe nature. Schizophrenia is a lifelong disorder that usually manifests after puberty and before 25 years of age, with equal risks across gender. The illness is episodic and places the sufferer at an increased risk of suicide.

Proteomics studies have focused extensively on cancer diagnosis and non-invasive monitoring, primarily via serum samples. Many have revealed potential biomarkers or biochemical molecules that identify a specific disease state and are capable of being detected or measured. For example, tumor marker CA125 (MUC16) provides useful information on disease resistance, treatment response, and even early detection in ovarian cancer screening, and efforts are underway for its clinical application [1].

Not surprisingly, the standards for viable biomarkers are high. Ideally, a diagnostic marker meets seven conditions: 1) it detects a fundamental feature of the disease with high sensitivity and specificity; 2) is validated in post-mortem confirmed cases; 3) standardized with sound bioinformatics; 4) specific for the disease compared with related disorders; 5) reliable in many testing environments/labs; 6) noninvasive; 7) simple to perform; and 8) inexpensive [adapted from [2]].

Despite the significant rates of self-harm behavior associated with schizophrenia, its relatively high prevalence in the general population and the existence of a substantial untreated population, no biomarker has yet been discovered for the disorder.

This article describes the ability of proteomic approaches to accurately identify novel biomarkers, which may provide a substantial insight into schizophrenia pathogenesis. In addition, proteomic investigations could lead to the discovery of diagnostic and prognostic biomarkers and potential new molecular targets for drug development. Class prediction methods may validate potential biomarkers, and class discovery may reveal distinct etiologies and subtypes for better categorization. Applying the same proteomic methods to clinically accessible fluids (e.g., serum, cerebrospinal fluid, and urine) would place invaluable objective analytical power in the hands of the clinician.

The clinical significance of basic science research in schizophrenia is effective biomarker discovery, efficient assaying techniques, a high level of statistical discrimination, and tailored drug development. However, such research also involves serious ethical considerations, particularly in the application of detection technologies. In addition, logistical challenges may impede the progress of clinical proteomics in schizophrenia.

## Genomics

Early investigations into the genetic component of schizophrenia utilized linkage studies as their primary analysis [see [3] for a review]. In pedigree studies, the location of many DNA markers were computed, compared in diseased and disease-free cohorts, and a probability of statistical significance (i.e., LOD score) was computed confirming or denying linkage. Meta-analyses of several linkage studies suggested correlations with some chromosomal regions, particularly 1q, 2q, 6q, and 11q; however, none approached an acceptable genome-wide significance [4]. While they have successfully mapped genes for monogenic or Mendelian disorders [5], linkage studies are inadequate for complex multi-factorial disorders like schizophrenia.

Later research employed association studies that compared allelic frequencies in schizophrenia and control groups. Essentially, it tested for linkage disequilibrium where two alleles or genetic markers are rarely separated by crossover. To minimize the ethnic variance on allelic frequency, family-based association studies were performed where at least one member was afflicted with schizophrenia. They revealed mixed results; however, the central areas where genetic aberration was mostly likely present, namely dopamine, serotonin, and NMDA receptors, showed little or no association [3].

Genetic studies have mapped many susceptibility loci as well as large chromosomal aberrations in schizophrenia [4,6,7]. However, the emergence of high-throughput microarray profiling allowed the rapid and economical assaying of thousands of gene expression levels. The prefrontal cortex, Brodmann area 9 (BA 9), was identified as a prominent site of dysfunction based on substantial neuroimaging, clinical, and postmortem studies, and has been the recent focus of microarray investigations [8,9]. Studies revealed the under expression of pre-synaptic markers [10], major metabolic pathways [11], and oligodendrocyte development and maturation genes [12].

Although genomic data may uncover novel information or corroborate current theories in schizophrenia pathogenesis, finding useful markers may be within the scope of proteomics. Gene expression data do not consistently correlate with protein expressions, and cannot identify post-transcriptional and post-translational modifications, major modulators of protein function, and presumably pathogenesis [13,14]. Moreover, the majority of schizophrenia microarray studies were based on post-mortem brain tissues, a clinically inaccessible medium.

## Proteomics

In the "post-genomic" era, the natural progression is towards interrogating the main effectors of physiological functions – proteins. The major genomic projects of the last decade have shaped proteome-wide sequencing, mapping, and analysis. For example, the creation of the Human Proteome Organization's Human Brain Proteome Project to foster the effective international communication of brain related proteomic data [15]. Complex diseases are now rapidly investigated by novel high-throughput biochemical inquires to uncover disease dynamics, clinical markers, and drug targets.

Contrary to the genome, the proteome is composed of an active array of molecules constantly being modified and with special localization. Proteomic approaches are able to characterize post-translational modifications, a method by which the cell dynamically and quickly modifies protein function and regulates both creation and degradation in response to cellular perturbations (e.g., disease provocation). Protein profiling and identification techniques using mass spectrometry (MS) and bioinformatics can lead to the discovery, identification, and characterization of protein biomarkers differentially expressed in the diseased states versus the control.

### Protein profiling

Similar to gene mRNA expression profiling, several protein profiling techniques emerged in the last decade that did not require *a priori *knowledge of candidate genes or proteins. From variations of gel electrophoresis to the advent of peptide specific mass spectrometry, each modality confers another method of differential protein expression analysis. Profiling the proteomes of diseased and healthy tissues allows for the discovery of peptide or protein molecular change, which potentially reveals information on pathogenesis or diagnosis, or both.

#### 2-Dimensional Gel Electrophoresis (2D-GE)

Initial proteomic studies relied on 2D-GE, which separates proteins based on two factors: isoelectric point and molecular weight. The process is complicated, burdensome, and certainly not robust. It was never intended to serve as a biomarker discovery wizard; rather, its use was limited to pathogenic discovery. Edgar et al. [16] applied this approach to the hippocampal proteome of schizophrenia and control groups to reveal 108 differentially expressed proteins. The most significantly under-expressed protein in their schizophrenic hippocampus was subjected to peptide digestion and N-terminal peptide sequencing. Based on a simple protein database search, their query revealed the protein to be diazepam-binding inhibitor (DBI). Reports indicate that DBI can bind to a GABA_A _recognition site and therefore down-regulate the action of gamma-aminobutyric acid (GABA), an inhibitory neurotransmitter system altered in schizophrenia [17,18].

Edgar et al. [19] further identified three differentially expressed proteins in schizophrenia: manganese superoxide dismutase (MnSOD) was under-expressed; and collapsing response mediator protein 2 (CRMP-2) and t-complex protein 1 (TCP-1) were over-expressed. MnSOD catalyzes the dismutation of superoxide anion (O_2_^-^) into water (H_2_O) and hydrogen peroxide (H_2_O_2_). In nervous tissue, it may protect the survival of cell membranes [20]. CRMP-2 regulated axonal growth and polarity [21,22]. Its over-expression may explain the vast neural interconnections in the schizophrenic brain.

TCP-1 is a chaperone protein that aides in proper protein folding and arrangement [23]. It has protective properties in the brain, preventing stress-induced apoptotic pathways in neurons [24]. 2D-GE analysis of fetuses with Down syndrome uncovered a significant decrease of TCP-1 in the second trimester, which may explain the disorder's early pathology [25]. However, it is over expressed in schizophrenia hippocampal tissue [19]. The authors suggested a possible alteration in cytoskeleton turnover in schizophrenia. Perhaps the mechanisms of a cytological aberration is by way of the post-translation modification – oxidation/nitration.

Oxidative stress is particularly implicated in neurodegenerative diseases like Alzheimer's [26] and Parkinson's diseases [27]. Gene expression studies with schizophrenia have consistently identified oxidation related transcripts. Theories have postulated that when the production of harmful oxidants exceeds the rate of anti-oxidant compounds, macromolecules such as DNA and proteins become the targets of oxidative attack, which signals subsequent death. Moreover, Japanese scientists were the first to identify proteins targets for nitration in the brains of rats, which included TCP-1 [28]. The original research methodology did not allow for post-translational modification testing; the state of TCP-1 in schizophrenic hippocampal tissue therefore remains unknown.

Chromosomal location is another feature of the study that sheds light on schizophrenia. Three of the four characterized proteins were mapped to chromosomal arm 6q. Their vicinity reveals a region important in schizophrenia and confirms the susceptibility of loci found by the linkage study [29].

Despite the informative studies using 2D-GE with schizophrenia, this modality proved very limited. Three important issues were raised that have instigated investigations into other resolution measures. First, 2D-GE analysis has limited reproducibility. Second, weakly soluble proteins cannot be easily resolved. Third, only a tiny portion of the proteome can be effectively stained. It is especially difficult for low-level expressed proteins and those masked by greater expression within a similar molecular weight or isoelectric point, or both.

#### Matrix Assisted Laser Desorption Ionization (MALDI)

The field of proteomics advanced dramatically with the advent of mass spectrometric (MS) analysis for peptides. There are four components to mass spectrometry. First, the *ion source *generates ionized peptides or proteins from the sample. Second, the *mass analyzer *sortes and resolves proteins based on their mass/charge (*m/z*) ratio. Third, the *ion detector *spots the ions and composes data on the ion *m/z*, quantity, and time of flight (TOF), or the time it took to reach the detector. Finally, *bioinformatic analysis *interprets the raw data into meaningful results (e.g., differential protein profiling).

MALDI is considered the standard of MS instruments. A special chemical matrix is applied over the sample and allowed to condense. After lasers activate the matrix, the energy is conferred to the peptides or proteins, and they are sent on a direct trajectory to the detector in a gas phase. This aids in two types of analysis: the creation of a peak pattern or signature specific to the disease, and in identification by inference. In the latter, the protein complex is usually proteolitically digested before ionization, creating specific peptide cleavages. After a MS run, and in a process called peptide mass fingerprinting, the peptide masses obtained from the *m/z *ratio are queried into several databases (e.g., UCSF's MS-fit and ExPASy's Aldente) to infer protein identification.

Jiang et al. [30] employed MALDI technology to analyze cerebral spinal fluid, and found significant down-regulation of apolipoprotein A-IV (apo AIV). However, given the paucity of published works correlating the CNS and this apolipoprotein, they were unable to extend their finding. Apo AIV is a glycoprotein secreted by the intestine [31], which signals the body's satiety after consuming lipids. In rats, apo AIV mRNA and protein were found in the hypothalamus and their concentrations correlated with feeding states [32]. The under-expression of apo AIV in schizophrenia suggests a correlation between the increased risk of weight gain and insulin resistance, either attributable to the disease or as a side effect of anti-psychotics [33].

Cerebral spinal fluid sampling presents a major challenge. Several highly expressed proteins, particularly serum albumin, transferin, and immunoglobulins, often mask lower abundance proteins. This study performed few purification measures to fractionate and better resolve the sample protein population. SELDI, a new generation of MS analysis, offers better resolution with built in chromatography. Researchers at Novartis in Switzerland have identified an optimized protocol for sample preparation and SELDI analysis for CSF studies [34].

#### Surface Enhanced Laser/Desorption Ionization (SELDI)

As with all spectrometric analysis, samples must be treated in some part before ionization. SELDI technology, a variation of MALDI, relies on ProteinChip arrays. Each chip offers a unique chromatographic surface for selective protein capture. For example, the IMAC ProteinChip incorporates an immobilized metal, often copper, as its vehicle for affinity capture. The CM10 ProteinChip is a weak-cation exchanger array, while the Q10 is a strong-anion exchange array.

Researchers at the ProteinChip manufacturer Ciphergen, in association with the NYU School of Medicine, applied SELDI analysis to schizophrenic postmortem prefrontal cortex brain tissue [35]. This approach sought to harvest spectral peak intensities and *m/z *values to develop signatures capable of differentiating schizophrenia from non-disease states. Using the three aforementioned surface chemistries, they maximized their total protein peaks to 1597. Forty-five peak differences were found to be statistically significant.

Class prediction using the decision rule algorithms where the informative peaks in the training set were used to perform leave-one-out cross validation. When incorporated into a statistical model from the training data set, the testing data set had a sensitivity of 69% and a specificity of 70%. In other words, 69% of schizophrenics were correctly identified using the signature, and 70% of normal individuals were appropriately recorded. These values fall short from cancer signatures in the range of 90–100% [36-40]. However, it affirms the heterogeneous etiology of schizophrenia and hints at the potential of SELDI methodology.

Nonetheless, SELDI technology has inherent limits. There can be competition between non-informative and informative peaks based on abundance and molecular weight. The proteins are competing to bind with the chromatographic molecules. Greater abundance proteins may conceal those with lower abundance. In addition, proteins with the same mass as the target protein may augment the mass intensity that is tested for.

### High-throughput protein identification & characterization

Studies that solely identify protein peaks for signatures or peak patterns do not provide information on schizophrenia pathogenesis and drug targets. Only after the identification of differentially expressed proteins can molecular mechanisms be unraveled. Moreover, if performed on a clinically inaccessible medium, such as brain tissue, they serve no function in the discovery of diagnostic biomarkers.

Although peptide mass fingerprinting is one method of protein identification, it often requires extensive and often complex purification, and it tenders an interpreted protein match by peptide masses rather than by sequences. Edman degradation, although offering amino acid data, is a slow task that cannot process N-terminally modified peptides, has limited sequence length (about 50–60 amino acids residues), and a low efficiency (e.g., missed cleavages).

#### Collision Induced Disassociation (CID)

MS has evolved to incorporate tandem mass spectrometric (MS/MS) technology that permits effective sequencing. The MALDI-TOF/TOF-MS technology is a cutting-edge proteomic utility with direct amino acid sequencing and characterization capabilities. Essentially, there are two TOF instruments separated by a fragmentation center, which allows for traditional MS for profiling and MS/MS signals for high-throughput identification.

The sample loading spots are subdivided into many fields and each field can be ionized independently. Moreover, they can be separately subjected to high-energy CID to induce fragmentation with optimized laser intensity and reflectron properties based on the protein molecular weight of interest [41]. Complementary fragments of the ion series are recorded when perceived by the MS detector. Data analysis software interprets uninterrupted high-energy CID fragment spectra into amino acid sequences. Identification of the protein is performed by querying the sequences against the protein databases (e.g., the NCBI nr) with database-searching algorithms (i.e., the probability based Matrix Science Mascot) [42].

As opposed to low-energy fragmentation methods, MALDI-TOF/TOF-MS is capable of high-energy CID, which can uniquely generate immonium ion fragments and cleave side chains of isobaric amino acids essential to distinguishing leucine and isoleucine. It yields unambiguous and reproducible sequence assignments vital to characterizing the proteins of interest.

#### Functional-complex study

No published schizophrenia study has yet employed the profiling and sequencing properties of tandem mass spectrometric analysis. However, several studies have demonstrated the potential of this technology in determining the complexities of the dynamic proteome. Sequence analysis can detect post-translation modifications such as acetylation, trimethylation, phosphorylation, sulfation, and N- or O-glycosylation [43,44]. It offers complex information such as ubiquitylation [45], which could reveal sites for repair, transcriptional regulation, and apoptosis.

This system can interrogate, identify, and characterize glycosylphosphatidylinositol (GPI) anchored proteins that are integrated into the plasma membrane [46]. The prion protein (PrP) is a prominent member of the GPI anchored protein family. Given that such proteins are involved in cell adhesion, differentiation, and host-pathogen interactions [47], it is a vital area of interest for schizophrenia. The Nogo-66 receptor gene (RTN4R) is a GPI anchored protein implicated in axonal growth inhibition, and a candidate for a schizophrenia susceptibility gene [48]. Even after proper isolation, these proteins are virtual inaccessible using conventional MS techniques. However, tandem technology with CID amino acid sequencing can recognize the protein's GPI attachment site (the Ω site), structure, and aberrations.

Reduced gene and protein expression of Reelin (RELN) [47] and up-regulation of neural cell adhesion molecule (NCAM) [49] is noted in schizophrenia. Recently, Eastwood and Harrison [50] confirmed reduced RELN mRNA expression in schizophrenic interstitial white matter neurons in the hippocampal formation and dorsolateral prefrontal cortex. Similarly, NCAM is over-expressed in the hippocampus and prefrontal cortex [51].

RELN is a secretory protease produced by GABAergic interneurons and binds to pyramidal neurons or GABAergic interneurons actively expressing the disabled-1 gene (DAB-1) product. Most of RELN's signaling pathways are mediated through DAB-1, including neuronal migration, synaptic plasticity, transmission, and survival. NCAM is a surface protein structurally similar to immunoglobulin and fibronectin. It is implicated in homophilic binding for cellular adhesion and the same processes as RELN.

These two glycoproteins are excellent targets for MS/MS characterization. Using affinity purification for the two molecules, the samples can be processed for protein complex identification using mass spectrometry. Protein binding partners can also be identified as a part of a complex when targeting known signal transduction domains (e.g., SH2 and Grb2).

This technique may reveal protein complexes within interaction networks. However, not only do dynamic post-translational modifications (e.g., ECAM) further complicate MS probing, background sample contaminants may produce false positives. Two-dimensional liquid chromatography systems can resolve composite protein mixtures based on chromatofocusing and reversed-phase chromatography [see [52] for further information].

#### Selective scanning by location

In the aforementioned studies using brain tissues, samples were homogenized and lysed, and the protein supernatant subjected to MS. Various cellular molecular profiles are pooled together losing descriptive spatial information and interfering with distinct profiling. Although the RELN study used immunohistochemistry and autoradiography to visualize its specific expression, those methods can only interrogate one protein per array and the user must have *a priori *information to perform them. However, to gain further insight into the molecular pathology of neuropsychiatric disease we can employ selective cellular proteomics.

One such technology, laser capture microdissection (LCM), isolates precise cellular, extra-cellular, and even sub-cellular areas for subsequent molecular analysis [53]. Pure populations of proteins (or other macromolecules) can be isolated under direct visualization using a microscope. Laser pulses transfer the biological material to a film. Downstream, the molecular concentrations are within the sensitivity of SELDI analysis.

Another medium, layered expression scanning (LES), has multiple membrane layers [54]. Biomolecules from tissue sections can be captured by each layer that retains its spatial orientation. There are two operating environments. In the open system, it can process macromolecules in a nonspecific fashion. In the closed system, membranes are pre-treated with an affinity binder (e.g., antibody) for individual protein localization; however, there can be numerous protein interrogations per a single tissue section.

While there are currently no published works applying these techniques to schizophrenic tissues, LCM and LES can provide substantial information about the pathogenesis of schizophrenia. Major brain regions, including the temporal lobe and executive function areas, are priority targets. Each of the six cortical layers can be independently examined per Brodmann area providing cellular level data on protein expression. Integrating the data in a bioinformatic platform to yield a validated biochemical model for schizophrenia is yet another realizable scientific feat.

## Clinical implications

Schizophrenia is a truly debilitating disorder that merits further proteomic inquiry. A PubMed search shows that of the 7,987 articles on the topic of proteomics, only 19 correspond to schizophrenia. In contrast, 1,339 were on cancer proteomics (the other papers were largely methodological reports). Regarding the limited findings already generated, and in anticipation of further studies that utilize a larger sample size, selective instrumentation, and quality controls, the problem lies in translating the data into clinically useful tools.

Translational research merges basic science and clinical research for optimal therapeutic benefit. The wealth of proteomic findings in the pipeline must be processed for clinical applicability to the effected patient population. To process clinical proteomics in schizophrenia where proteomic strategies are married to the discipline of medicine, we must understand the disease profile, etiology and pathogenesis, diagnostic strategies, and finally clinical management. In each medical realm, proteomics has a unique and revolutionizing role.

### Clinical profile

Clinical symptoms of schizophrenia usually begin in late adolescence or early adulthood. They are generally grouped into three broad categories: "positive" or "negative" based on the pathological effects of normal functions, and "cognitive impairments." Over time, positive and negative symptoms tend to be episodic and to vary in intensity.

#### Positive symptoms

These include symptoms that are considered exaggerations or distortions of normal functions: psychosis, false beliefs (delusions; 90% incidence in all subjects), perception of something when nothing in fact exists in the perceptual field (hallucinations; 50% incidence), and bizarre behaviors [55].

#### Negative symptoms

Negative symptoms are deficit states in which fundamental emotions are either weakened or entirely deficient, including blunted affect, anhedonia (inability to experience pleasure from normal activities), apathy (loss of interest and motivation), social withdrawal, and alogia (diminished speech content). They have an earlier and more subtle onset, and are less episodic than psychotic symptoms.

#### Cognitive symptoms

Schizophrenia may encompass disturbances in cognition, usually related to attention and concentration, learning and memory, psychomotor speed (e.g., prolonged reaction time), and executive processing (e.g., formulating and initiating plans, abstract thinking, and problem solving).

Schizophrenia is a devastating psychotic disorder because it destroys the social functioning and employability of patients. Negative symptoms and cognitive impairment are generally the disabling mechanisms of schizophrenia. A patient may no longer have the ability to concentrate on and take pleasure from work, studies, or leisure activities. Moreover, the patient's lack of medical insight further hinders their ability to take advantage of effective coping strategies, which, in turn, can further aggravate social withdrawal, depression, and the risk of suicide.

### Etiology

Schizophrenia is a multifaceted disorder manifested by both genetic and environmental factors. A plethora of twin and adoption studies have suggested major genetic influences on the pathogenesis of schizophrenia; however, a MZ concordance of about 50% [56] also indicated the involvement of environmental factors. These family studies reveal that schizophrenia is a complex genetic disorder, akin to diabetes and cancer, with not one causative gene but rather multiple genes contributing to susceptibility. In other words, it is a polygenetic disease. Moreover, there are presumably environmental factors that contribute to the onset of the disease.

The *vulnerability-stress-coping model *frames psychotic and affective disorders from a biopsychosocial perspective [57-59]. Vulnerabilities may predispose the individual to the disorder, while environmental stressors can potentially modulate (trigger) the expression of symptoms in vulnerable bodies-minds.

In schizophrenia, *vulnerability *may include genetic predisposition, birthing complications, and CNS viral infections. Stressful life events (e.g., being fired from work, terminating a relationship, or moving into a new environment) and biological *stressors *(e.g., substance abuse) may exacerbate the illness by triggering the emergence or reoccurrence of symptoms. However, protective *coping *mechanisms can safeguard vulnerable persons by weakening or eliminating symptoms.

The *vulnerability-stress-coping model *demonstrates the composite mechanism of schizophrenia and provides a useful diagram for optimal combination therapy for clinical management. However, it does dramatically complicate any proteomic screening. Studies using samples from tissue collections (e.g., the Stanley Brain Research Laboratory and Brain Collection) risk skewing their findings by factoring in confounding drug, storage, and various *stressor *effects.

### Pathogenesis

Despite extensive efforts through histological, neurochemical, neuroimaging, and gene and protein expression studies, the biomedical community has yet to uncover a definitive diagnostic neuropathology for schizophrenia. However, several important findings do collectively direct our knowledge of schizophrenia pathology and contribute to medical interventions. Initially they were directed by pharmacologic manipulation; however, there has been progress towards uncovering genetic biological markers using genomic and proteomic strategies. They have progressed the understanding of schizophrenia from psychosomatic origins to a brain disease subject to objective study.

At present, the medical community is familiar with pathological alterations of the dopamine (DA), serotonin, acetylcholine (ACh), and glutamate systems. Studies into the use of substances that induce psychosis (e.g., amphetamines) have revealed enhanced reuptake of DA [60]. These findings initiated the dopamine hypothesis, which states hyperactive DA transmission in schizophrenia, perhaps in response to stress. The brain is essentially overly sensitive (hyperactive) to stimuli and fails to properly regulate its response through normal inhibitory mechanisms. To potentially explain negative symptoms, brain imaging studies have revealed prefrontal cortex (PFC) dysfunction [61,62], specifically a regional deficit in DA neurotransmission [63].

The current view of the dopamine hypothesis relates hyperactive subcortical and hypoactive cortical DA neurotransmission to positive symptoms and negative-cognitive impairments, respectively. However, the theory is contested. For example, the total inhibition of DA does not fully mitigate the positive symptoms of schizophrenia [64], suggesting additional pathological disturbances.

Recent genomic and proteomics studies have moved away from a purely disrupted DA model to that of oxidative stress and synaptic pathology, which may cause dysregulation of several neurotransmitters and neuronal apoptosis [see [65] for a review]. Various presumed susceptibility genes and their products have been identified: neuregulin-1 (NRG1), dysbindin (DTNBP1), regulator of G-protein signaling 4 (RGS4), catechol-o-methyltransferase (COMT), proline dehydrogenase (PRODH) and disrupted-in-schizophrenia 1 (DISC1) [66]. However, there are clinical applicability issues with diagnostic specificity [67] and the small effect size [68].

### Diagnosis

Given the lack of an objective and definitive diagnostic test, clinicians currently look beyond the organ and disease-specific approach in psychiatric diagnosis. Clinicians interview patients to watch for abnormal behaviors, ascertain risk factors, and record a personality profile. The clinician actively listens to the patient and establishes a comfortable atmosphere to reveal pertinent information.

Diagnostic classification manuals such as the American Psychiatric Association's Diagnostic and Statistical Manual of Mental Disorders (DSM) [69] serve as provisional constructs allowing the international biomedical community to employ inclusion and exclusion criteria based on potential deviations from normal psychological functioning. In fact, they explicitly serve not to educate about a particular etiology or pathology; rather, they reasonably classify disorders by symptom profiles for effective universal communication. Manuals essentially offer standards for symptom definition and differential diagnosis. Nonetheless, they may create false perceptions of known discrete disorder entities that are far from the multifarious and overlapping reality.

#### Current criteria

Current DSM diagnostic criteria for schizophrenia include at least one month of active symptoms (at least one of bizarre delusions or auditory hallucinations, or two or more individual positive and/or negative symptoms) and a six-month period of social/occupational dysfunction or independent-care impairments. Patients may have brief psychotic reactions for one to six months that resemble schizophrenia, known as *schizophreniform disorder*, but rapidly remit and do not reoccur. The diagnosis, however, may in fact be mood disorders with psychotic features.

In addition to inclusion principles, the DSM establishes exclusionary guidelines for primary mood disorders with psychotic features (e.g., unipolar or bipolar depression) and psychosis-induced by the physiological properties of a general medical condition or chemical substances.

#### Classification

The current scheme of schizophrenia classification, like for other psychiatric disorders, stems from the clinician's observation of gross behaviors. Consequently, it is rather imprecise and does not necessarily correlate with genetic pathology. A movement for molecular class discovery in complex disorders is in place where genetic observations can subtype or differentially cluster groups based on gene or protein expression or susceptibility genes or their products. As opposed to the class prediction or supervised learning where knowledge of the conditions are known to yield an expression signature, class discovery or unsupervised learning uncovers patterns with no prior knowledge of traits and assembles clusters. For example, extensive phenotypic characterizations and diagnostic groups were identified for inflammatory bowel disease, permitting productive genetic analysis [70]. In addition, supervised and unsupervised analysis was applied to leukemia DNA microarray data [71]. It demonstrated an ability to distinguish between acute and chronic leukemia and a special gene subset for leukemia forms.

By recognizing the imperfect relationship between genotype and phenotype in psychiatry, Gould and Gottesman [72] anticipated the discovery of *endophenotypes *to improve genetic studies. In a reductionist approach, this classification is a simplified measure for a single neuronal circuit with fewer genes or proteins. Five criteria were identified for an endophenotype: 1) it is associated with illness in the general population; 2) heritable; 3) state independent (regardless of illness activity); 4) cosegregates with illness in families; and 5) the endophenotype identified in probands is found in unaffected relatives at a higher rate than in the general population.

Given the spectrum of neuronal circuits implicated to date, a complicated disorder like schizophrenia is presumably composed of multiple endophenotypes. Studies employing this approach create more homogenous subtypes, rather than merely altering their defining observations. For example, *working memory *is considered an endophenotype in schizophrenia [73]. They found a population of schizophrenia patients had a significantly disturbed working memory and demonstrated that it was a partially inheritable deficit. Furthermore, it can be assessed using conventional tests (e.g., Wisconsin Card Sorting, backward and forward digit span, and digit symbol substitution).

Applying powerful class discovery to schizophrenia in association with specific endophenotypic descriptors may reveal signatures and true classification strata. Preliminary data from Bowden et al. [74] indicated the ability to subgroup schizophrenia by age using gene expression data from peripheral blood lymphocytes. Appling this to mode of death, duration of illness, medication, drug use, alcohol abuse, smoking patterns, genetic relative risk, and related classifiers provides valuable information. However, applying it to specific schizophrenia endophenotypes could shape validated classifications, more precisely identify pharmaceutical targets, and prove immensely important in the future of schizophrenia molecular biology studies.

#### Differential diagnosis

The development of biomarkers capable of differential diagnosis would be a paramount achievement in medicine. In clinical practice, it is essential to uncover the underpinnings of psychosis, discover the etiology and pathology, and then design and implement sound clinical management. Whether the disorder is schizophrenia, affective, or organic in nature plays a significant role in determining the proper treatment. Patients that present with disturbed behavior and/or mentation may be hurriedly and incorrectly deemed psychiatric, when in fact the psychotic symptoms are secondary to general medical conditions or substance abuse. Moreover, mood disorders may present with poor reality testing but require treatment of the mood disturbances and psychosis; however, they are difficult to differentiate from schizophrenia.

Central nervous system stimulant and depressant-induced intoxication and withdrawal may encompass psychotic features that complicate diagnostic efforts. Chronic abuse of psychoactive substances, such as amphetamines and cocaine, stimulate dopamine neurotransmission and trigger hallucinations and delusions. Users of phencyclidine (PCP) may experience psychosis, agitation, and violent behaviors [75]. It is imperative to run toxicology blood tests, sometimes beyond the standard drug screens, based on clinical observations.

When proteomic studies implicate two testing groups – schizophrenia and control – their biomarker findings may represent biological compounds with similar altered expression patterns in related disorders. This is especially pertinent for disorders with symptoms stemming from a common etiology. For example, serum studies in schizophrenia that isolate inflammatory markers may have similar findings to disorders with inflammation (e.g., in reference to differential diagnosis, bipolar disorder, Alzheimer's disease, Parkinson's disease, head trauma, and brain tumors).

Examining samples from patients with disorders of common and exclusive endophenotypes provides the best insight into each disorder component – the factor that the clinical will treat. For explanation purposes, let us hypothetically start with multiple endophenotypes. Biological samples from individuals with schizophrenia, bipolar disorder, major depressive disorder, and normal controls are examined by protein profiling. In each diseased group, sub-populations are defined based on individual assessments of each endophenotype. Class prediction may identify protein peaks that discriminate the various groups based on disease classification. However, analysis with endophenotype descriptors may reveal markers that best correlate with endophenotypic dysfunction and thus the treatment target.

### Clinical management

The ultimate goals of schizophrenia clinical management are to reduce or eliminate all associated symptoms, improve socio-behavioral functioning, foster reintegration into society, prevent the relapse of psychotic episodes, and treat or prevent (further) co-morbidity. Clinicians employ a combination of pharmacological (neuroleptic) and psychosocial interventions according to the *vulnerability-stress-coping model*. The first line of coping is usually medicinal options for the suppression of symptoms and control of disturbed behavior. Psychosocial modalities contribute to improve patient insight and compliance, while promoting the development and implementation of personal goals.

Since the 1950s and until recently, first-generation antipsychotic medications have been the staple of schizophrenia treatment (e.g., chlorpromazine and haloperidol). The neuroleptic mode of action is presumably DA blockade in the mesolimbic-mesocortical system – DA receptor antagonism [76]. However, if the antipsychotic action and the antischizophrenic action were one and the same, schizophrenic symptoms would cease just hours after administration. This is not the case; rather, gradual improvement is observed over a period of weeks. The antischizophrenic properties of neuroleptics may be attributable to a tolerance mechanism caused by long-term therapy. Essentially, DA neurotransmission returns to a more normal state, significantly improving positive symptoms.

Most patients taking first-generation antipsychotic agents have some response, with 20% experiencing complete remission [77-80]. However, neuroleptic-induced DA blockage in the basal ganglia and more generally the nigrostriatial system contributes to extrapyramidal reactionary movement disorders [81].

Over the past 15 years, clozapine has served as the progenitor of second-generation (atypical) antipsychotic drugs. All second-generation drugs share the DA blockade mechanism of the first-generation; however, they have selective affinity for DA and similar receptors, and also implicate additional neurotransmission systems (e.g., serotonin). Clinically, their efficacy is established by reducing extrapyramidal side effects, and therefore improving medication compliance, treating refractory schizophrenia, and reducing negative symptoms and cognitive impairments. However, clozapine is never prescribed as the first antipsychotic because it presents with an increased incidence of seizures and agranulocytosis – an immune system disorder marked by a decrease of granulocytes – and thus patients are prone to chronic bacterial infections [82].

Newer second-generation drugs, such as risperidone, olanzapine, quetiapine, and ziprasidone, have at an equal or greater clinical efficacy and better adverse effect profile than first generation drugs, and a lower incidence of agranulocytosis than clozapine [83,84]. All neuroleptics operate to reduce positive symptoms; however, second-generation drugs have a greater effect on negative symptoms and cognitive function.

#### Drug discovery

Drug discovery for psychiatric disorders has been largely haphazard to date; however, a systematic approach is progressing. The procedures for drug discovery in schizophrenia include target discovery and validation, dose selection, clinical end points, and responder identification. First, two types of biomarkers are identified: disease-specific and drug activity markers. The former characterizes the disease, and the latter monitors drug interactions. Second, toxicity prediction is implemented for dose selection. Third, surrogate end points are used to predict important clinical outcomes. For example, blood pressure is a surrogate outcome for a stroke. Substituting clinical end points (e.g., survival) for surrogate end points (e.g., lowered blood pressure) allows for a reduced sample size yet maintains statistical power and a shorter patient monitoring term. Finally, the study identifies the patient populations that have received the intended benefit.

Proteomics has the ability to significantly affect drug discovery in schizophrenia by three principle means. First, protein profiling and identification techniques may identify novel pharmaceutical targets and co-regulated compounds. Second, proteomics can measure current drug responses. In effect, it can aid with efficacy and toxicity by screening animal models. Finally, it can function as a means of surrogate end point only if the compounds in question have a true association to mortality or the morbidity in question.

Unfortunately, the very disease symptoms that demand enhanced drugs – cognitive and negative symptoms – do not have proper animal models. Chen, Lipska, and Weinberger [85] demonstrated the impossibility of trying to recapitulate the full spectrum of schizophrenia in animal models. Although schizophrenia-like behaviors were shown in hypothesis-driven genetic mouse models, particularly via disrupted DA or glutamine neurotransmission, cognitive ability and emotional intelligence is difficult to test in animal models. Nonetheless, neuro-electrophysiology is a promising tool for clinically testing schizophrenic patients and animal models with schizophrenia-like abnormal information processing [86].

## Ethical considerations

The prospect of genetic testing for schizophrenia has generated a range of ethical, legal, and social issues that must be considered. A vital part of schizophrenia research is the protection of vulnerable individuals from unreasonable risk and to prevent the exploitation of research participants. The US Department of Health and Human services has established a regulatory code for the *protection of human subjects*. However, as a set of guidelines, they do not specifically bar certain controversial lines of schizophrenia testing. That naturally leads to ethical quandaries concerning the ability of *mentally disabled persons *to offer informed consent, and the impact of withdrawing drug treatment or permitting drug-naïve patients to participate in biomedical research. These topics have been discussed at length elsewhere [87-89]. The discussion below briefly focuses on issues involving the use of psychiatric biomarkers for eugenics and sterilization and as an employment screening tool (e.g., disqualifier).

### Reproductive freedom

Contemporary Western bioethics considers human dignity to be intrinsic to all human individuals [90]. In the United States, beliefs and attitudes associated with mental illness have dramatically changed with the understanding of biological pathogenesis and the implementation of combination treatment options. However, a survey has revealed a general negative perception of individuals with genetic disabilities among international geneticists, as well as government involvement in premarital testing and sterilization in China [91].

China has been under intense scrutiny since the passage of the Maternal and Infant Health Care Law in 1995. Although it prohibits prenatal genetic diagnosis for the purpose of sex selection, it does permit the use of eugenic statutes for the systematic abortion of offspring suspected of having serious medical disorders, including mental illness [92]. Furthermore, the law authorizes the sterilization of genetic carriers. China could conceivably apply these provisions to any genetic susceptibility found for schizophrenia.

Chinese scientists must be made to realize that their studies will not be published, access to foreign funding restricted, international collaboration halted, and the nation's reputation jeopardized if these eugenics policies are not abolished and legally binding and effective monitoring systems are established for biomedical research. Otherwise, the fruits of genomic and proteomic investigation could lead to the reproductive suppression of the very individuals we are attempting to understand and treat.

### Occupational discrimination

The development of diagnostic technology in schizophrenia could also be used as a tool for occupational screening. Selection for jobs that require exceptionally specialized training (e.g., astronauts) could eventually involve genetic screening for schizophrenia. While Presidential Executive Order (13145) prohibits the discrimination of federal employees based on genetic information, certain exceptions could permit this practice (e.g., interference with job duties). Quick [93] has argued the need for federal legislation given the highly variable application of laws in state court cases. Currently, thirty-one states ban workplace genetic discrimination [94].

This screening and potential genetic discrimination could even extend to intellectual positions (e.g., medical doctors). Studies have shown that employers are increasingly making use of genetic testing [95]. We need to fill the current void with professional guidelines that frame workplace genetic screening. Conceivably, it may implement voluntary screening procedures, a system of informed consent, and mutually agreed outcomes.

## Conclusion

Schizophrenia is associated with greater co-morbidity and lowered life expectancy. The disease process, antipsychotic interventions, and socioeconomics foster very unhealthy habits (e.g., poor diet, smoking, drug abuse) and disease (e.g., obesity, diabetes, hyperlipidemia, cardiovascular disease, cataracts). These factors contribute to an overall decline in health, interfere with clinical management, and place a strain on the medical infrastructure. Scientists and their funding sources are realizing the potential impact of psychiatry genomics and proteomics for relieving the burdens associated with major psychiatry disorders.

Proteomic utilities have just begun to define schizophrenia with molecular biology detail. Conventional modalities in proteomics have restrictive limitations, preventing the interrogation of proteins under a certain molecular weight (e.g., the majority of neuropeptides), those found in or associated with the plasma membrane (e.g., GPI anchored proteins), and the post-translation modifications heavily employed in brain tissue. Ultimately, technology provides insight into only a small portion of the proteome. However, advances in mass spectrometric measures, namely tandem MS technology, offer the potential to identify and characterize novel biomarkers.

Unlike cancer proteomics, schizophrenia proteomics faces several obstacles before it can be used in laboratory medicine. We must adopt an integrative approach to studying schizophrenia in order to generate proper classifications (e.g., endophenotypes and unsupervised class discovery), distinguish causality from mere association (e.g., putative susceptibility versus true biomarkers), utilize quality control measures (e.g., standardize practices and variation measurements), and take a proactive stance on the improper use of schizophrenia genetics.

Schizophrenia remains a disabling condition, but progress in the genomic and proteomic sciences, more efficacious pharmacology for negative symptoms and cognitive dysfunction, and greater societal awareness and appreciation of mental health issues may help alleviate this problem. The discovery of schizophrenia biomarkers will likely revolutionize the field of psychiatry, as did the introduction of psycho-pharmaceuticals, but such progress is still substantially in the future.

## Competing interests

The author(s) declare that they have no competing interests.
